# Coastal erosion—a “new” land-based source of labile mercury to the marine environment

**DOI:** 10.1007/s11356-018-2856-7

**Published:** 2018-08-10

**Authors:** Urszula Kwasigroch, Magdalena Bełdowska, Agnieszka Jędruch, Dominka Saniewska

**Affiliations:** Institute of Oceanography, University of Gdańsk, Piłsudskiego 46, 81-378 Gdynia, Poland

**Keywords:** Mercury fractionation, Mercury load, Coastal erosion, Cliffs

## Abstract

Mercury (Hg) can be introduced into the marine environment in many different ways. In the case of the Baltic Sea, rivers and atmospheric deposition are the predominant ones. However, in the face of ongoing climate change, a new potential source, coastal erosion, is starting to become more important and is currently considered to be the third largest source of Hg in the Gdansk Basin region. It is especially significant along sections of coastline where, due to the higher frequency of extreme natural phenomena such as storms, heavy rains, and floods, increased erosion processes have already been noted. Cliffs, which account for about 20% of the Polish coastline, are particularly vulnerable. The aim of the study was to estimate the annual load of labile Hg entering the Gdansk Basin as a result of coastal erosion. Samples of down-core sediments (0–65 cm) were collected in the years 2016–2017 from selected cliffs situated in the Gulf of Gdansk area. The thermodesorption method was used to distinguish between labile and stable fractions of Hg. Considering the mean total Hg concentrations in the collected sediments (9.7 ng g^−1^) and the mean share of labile (64%), bioavailable mercury, it was estimated that the load of labile Hg originating from coastal erosion entering the Gdansk Basin is 10.0 kg per year. The load can increase by up to 50% in the case of episodic abrasion events during heavy storms and rains.

**Electronic supplementary material:**

The online version of this article (10.1007/s11356-018-2856-7) contains supplementary material, which is available to authorized users.

## Introduction

Mercury (Hg) is one of the most dangerous environmental pollutants. This is primarily associated with its toxicity, which depends on the chemical form. The organic forms of Hg (i.e. methyl mercury—MeHg) formed in the methylation process are the most dangerous for organisms (Hong et al. 2012). Even low Hg levels in the body can lead to irreversible damage to the brain and the nervous system, as well as to the disruption of hormonal and enzymatic reactions. Hg penetrates through the placenta barrier, causing miscarriages and foetal defects (Bose-O’Reilly et al. 2010; Gibb and O’Leary 2014). For people who are not occupationally exposed to Hg, the main penetration route of the metal into the human body is the consumption of fish and seafood (Hong et al. 2012; Kalogeropoulos et al. 2012). It is related to the fact that marine organisms are characterised by elevated concentrations of Hg (and in particular MeHg). The Hg level in their tissues exceeds the values measured in the surrounding environment by several orders of magnitude. Hg concentration also increases with the trophic position of the organism—the highest Hg level is recorded in top predators (Fitzgerald et al. 2007; Mason et al. 2012). Therefore, it is particularly important to study the inflow and transportation processes of Hg in the marine environment.

Hg enters the sea mainly via rivers transporting pollution from the catchment area and through atmospheric deposition (Bełdowska et al. 2014). For coastal areas, an important role is also played by Hg washed out of land as a result of coastal erosion. The research carried out by the authors in previous years showed that the share of this source in the Hg load to the Gulf of Gdansk is over 5%, which makes coastal erosion the third most important route of entry for Hg to the basin—after rivers and wet atmospheric deposition (Bełdowska et al. 2016). The share of coastal erosion in the Hg load introduced into the marine environment may, however, be significantly higher in regions with limited riverine inflow. In the case of the Puck Bay, with only small rivers flowing into it, the share of coastal erosion in the Hg balance was as much as 33% of total Hg load (which corresponds to 14 kg a^−1^) introduced annually into the sea, indicating that coastal erosion is an important source of Hg to the marine environment (Jędruch and Bełdowska 2014). The significance of this source in the marine Hg cycle may increase over the next few years—it is related to the forecast intensification of extreme natural phenomena such as storms, downpours, and floods (Kożuchowski 2009; HELCOM 2013; Bełdowska 2015). This means that considerable loads of eroded sediment material (mainly from cliff sections) may be introduced into the marine environment along with the Hg accumulated in it. Previous studies by the authors showed a relationship between incidents, where a considerable part of the shore crumbled into the sea and an immediate occurrence of increased Hg concentrations in suspended matter and in phytoplankton—the first link in the trophic chain (Bełdowska et al. 2016). The increase in Hg concentration in phytoplankton indicates that at least some of the Hg in cliff sediments occurs in a bioavailable form for organisms and may, therefore, be bioaccumulated and transferred to higher trophic levels, reaching elevated concentrations in fish and shellfish consumed by humans (Fitzgerald et al. 2007; Mason et al. 2012). This is particularly important in the marine coastal zone, where fauna and flora thrive, accumulating pollutants from land (Bełdowska 2016; Bełdowska et al. 2016a; Staniszewska et al. 2016; Jędruch et al. 2018b).

The purpose of the present study was to determine the share of individual Hg forms—potentially bioavailable labile and stable forms—in eroded sedimentary material from selected cliff sections, as well as to assess the load (kg a^−1^) of particular forms of the element introduced annually into the marine environment, as exemplified by the Gdansk Basin.

## Materials and methods

### Study area

**Fig. 1:**
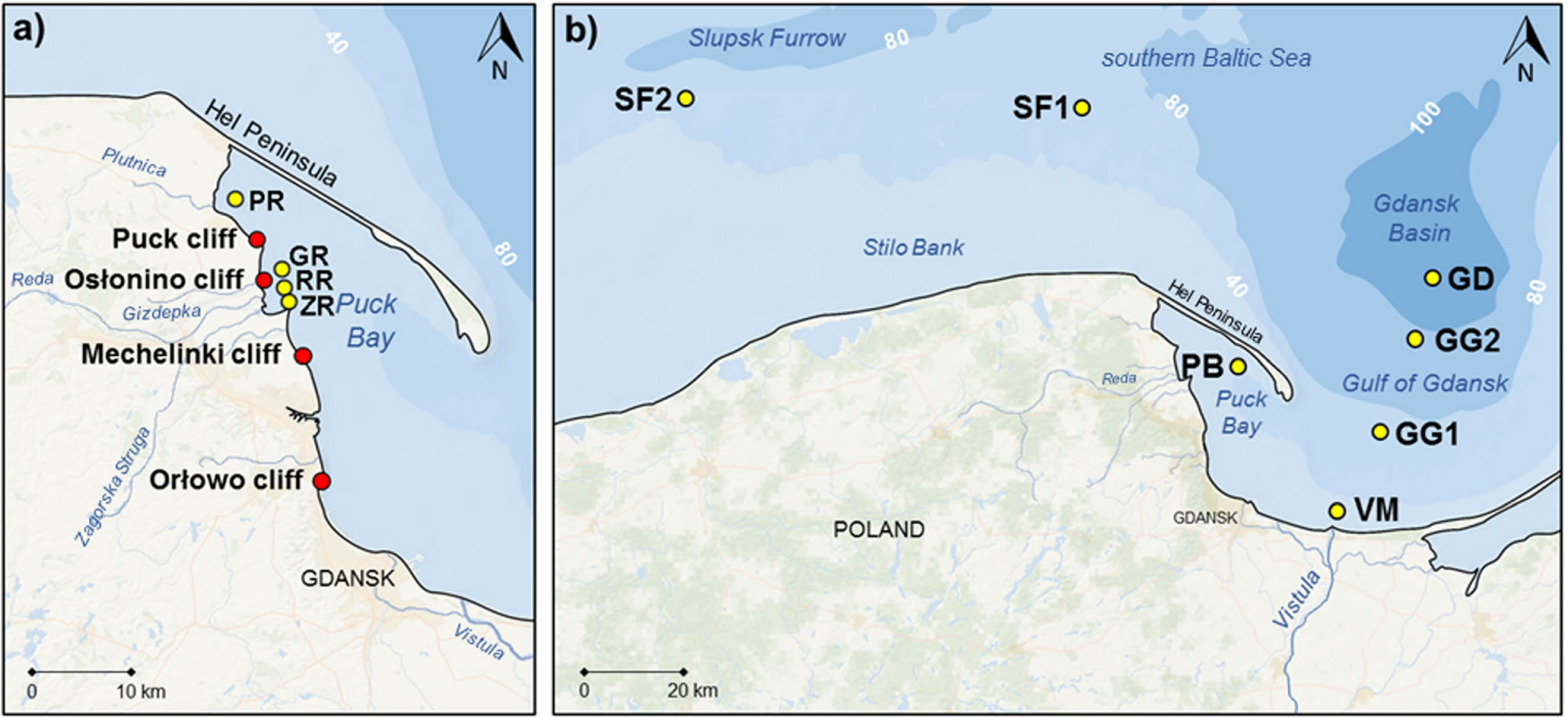
Location of sampling stations in the southern Baltic Sea region. **a** The cliff sediments (red dots). **b** Marine sediments (yellow dots)

The research was conducted in the area with four cliffs located on the west part of the Gulf of Gdansk, in the southern Baltic Sea (the Orlowo, Mechelinki, Oslonino and Puck cliffs in Poland) (Fig. 1a). These cliffs were created in postglacial moraine deposits, which were eroded by the sea during Littorina Transgression (Harff et al. 2017). Cliffs, occurring in segments measuring between 0.5 and 10 km (Subotowicz 1982), are scattered along almost the entire 500 km of the Polish coast and currently take up about 101 km (20%) of its length (Uścinowicz et al. 2004). They are characterised by a great diversity in terms of geological structure, height and vegetation coverage. The geological structure characterised by rock types and their interbedding, as well as the inclination of particular layers, are the key elements affecting the rate of erosive changes occurring in the region and the shape of the cliff wall (Łabuz 2013). The main materials comprising the studied cliffs are quaternary clay glacial formations, as well as fluvioglacial sands and gravels (Subotowicz 1982). Younger geological formations such as peats, aeolian sands and ice-marginal sands are far less commonly seen (Subotowicz 1980; Uścinowicz et al. 2004). The main constituent of the Polish coastal deposits is silicon (SiO_2_)—it occurs mostly as quartz and silicates. The silicon content in the sediments strongly depends on their grain size, reaching the highest content in sands. Coastal deposits are characterised by high concentration of iron (Fe_2_O_3_) in different mineral forms, represented by magnetite, haematite, biotite and glauconite. It is similar in case of manganese which is present in relatively high concentrations (mainly MnO), even in sands and silt-clay sediments. In sandy deposits, the occurrence of manganese is associated with ilmenite (Szczepańska and Uścinowicz 1994; Uścinowicz and Sokołowski 2011). It is crucial because the content of Fe and Mn oxides strongly determines the sorption of Hg in sediments (Pempkowiak 1997). Deposits are also characterised by lack or very low content of calcium carbonate (CaCO_3_) what is typical for fluvioglacial Pleistocene sands (Uścinowicz and Sokołowski 2011). In the case of minerals containing Hg, such as cinnabar or calomel, they have not been reported in the research area (Karwowski and Szełęg 2005).

The Orlowo cliff (Fig. 1a) is the most active part of the Gulf of Gdansk shore—there are precipitous slopes of a loose nature, landslides and sagging cones. The length of the Orlowo cliff is 650 m, and the height varies from 10 up to 50 m in the central part. In its composition, it is possible to identify two levels of boulder clay from the Middle Polish Glaciations and Vistulian Glaciation, interspersed with loam with sand interbedding of the fluvioglacial type (Łęczyński and Kubowicz-Grajewska 2013). The Mechelinki cliff (Fig. 1a) is mostly made of boulder clay, in which two layers can be distinguished: grey and brown clay of 13 and 9 m in thickness, respectively. The Oslonino cliff (Fig. 1a) is a 400 m segment of the coast in the Puck Lagoon area, also composed mainly of boulder clay with a small share of fluvioglacial sands and gravels in the northern part. The Puck cliff (Fig. 1a) is characterised by a very similar geological structure, with the predomination of brown glacial clay. The activity of the last three cliffs is much lower, and this is closely related to the protective nature of the Hel Peninsula (Zawadzka-Kahlau 2012; Łabuz 2013).

### Sample collection

**Fig. 2:**
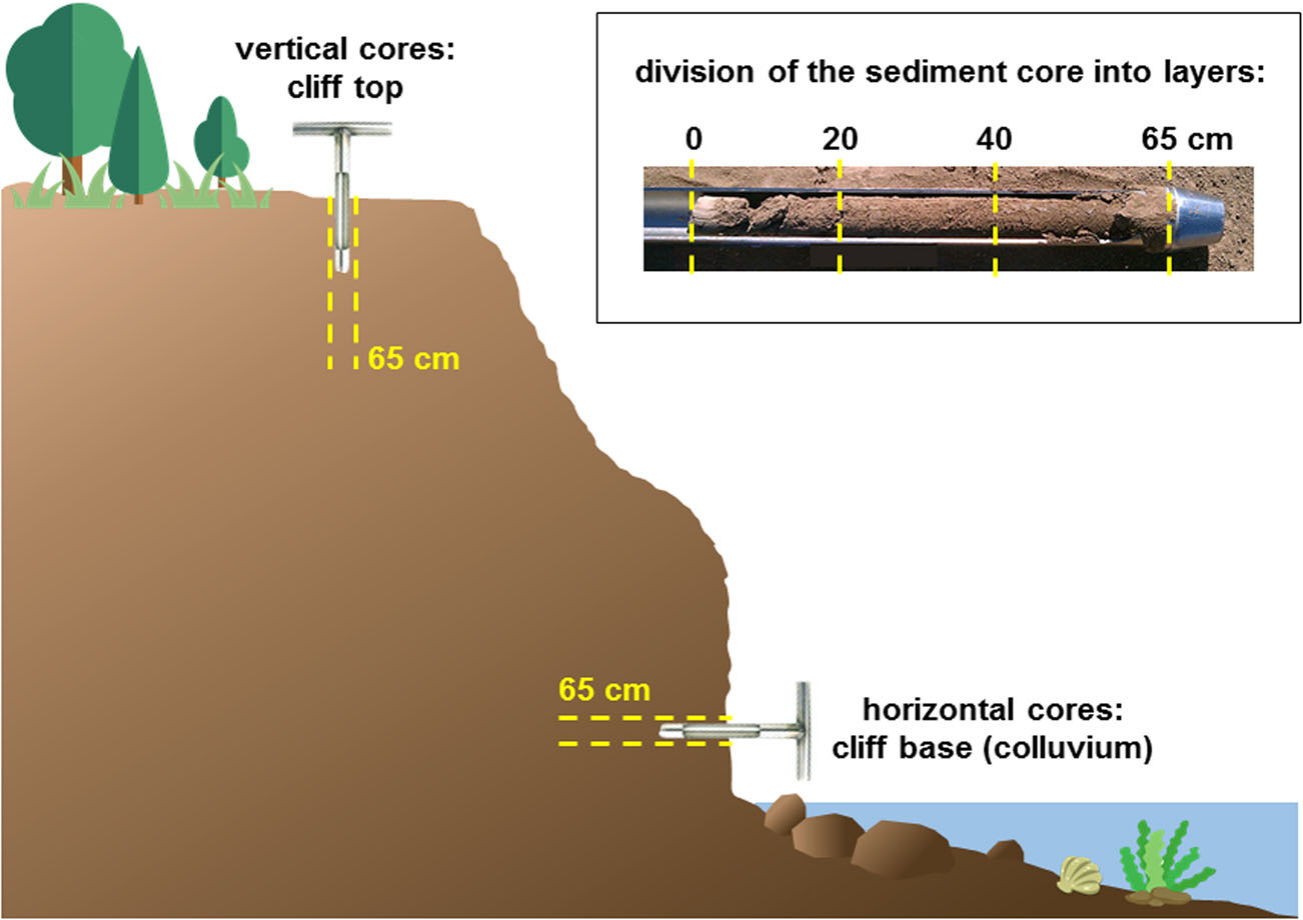
Scheme of sediments cores collection from the colluvium and the top of the cliff

The research was carried out from June 2016 to March 2017. On each cliff (Fig. 1a), three stations were set, located about 50 m apart from one another. In the area of the three studied cliffs (the Mechelinki, Oslonino and Puck cliffs), vertical and horizontal cores (0–65 cm) of sediments were collected. The horizontal cores were taken from cliff walls at a height of approx. 2–3 m. The vertical cores were taken from the cliff tops (Fig. 2). The sediment cores were collected using a soil core sampler (AMS, Inc., USA). At the Orlowo cliff, only the sedimentary material within the colluvium from the base of the wall and the surface sediment layer (about 20 cm) from the upper part of the cliff were collected. This was due to the fact that this section of coastline is part of a nature reserve (Kepa Redlowska), and invasive core sampling is not allowed. In addition, in the same four regions designated on each cliff, sediments from the beach and marine sediment from the coastal zone at a depth of about 1 m (5–10 m from the shore) were also collected. Additionally, samples of surface marine sediments were collected at research stations, differing in terms of environmental conditions (i.e. water temperature, salinity, density) (Fig. SI) and sediment characteristics (Uścinowicz 2009). The seven sampling sites were located in the southern Baltic Sea: in the Vistula mouth (VM) (depth 16 m; distance from the shore about 5 km), in the semi-enclosed Puck Bay (PB) (depth 35 m; distance from the shore about 12 km), the central part of the Gulf of Gdansk (GG1 and GG2) (depth 70 and 89 m; distance from the shore 20 and 38 km, respectively) and the Gdansk Deep (GD) (depth 105 m; distance from the shore about 50 km) as well as from the open waters of the southern Baltic Sea: the Slupsk Furrow (SF1 and SF2) (depth 65 and 68 m; distance from the shore 34 and 55 km, respectively) (Fig. 1b). Samples of marine sediment were collected from the R/V Oceania, using a van-Veen grab sampler during the cruise in May 2016 and additionally, the samples of surface sediments from outlets of small rivers that flow into the Gulf of Gdansk, near the studied cliffs: the Gizdepka river (GR), the Reda (RR), the Plutnica (PR) and Zagorska Struga (ZR) (Fig. 1).

All activities related to sampling were carried out in accordance with clean techniques protocols, required to minimise the sample contamination (i.e. “clean hands, dirty hands” technique, non-metallic sampling equipment, powder-free gloves), according to the guidance described in US EPA 1229 (US EPA 1996) and 1631 (US EPA 2002) methods. All samples were collected and shipped to the laboratory individually double-bagged to prevent potential cross-contamination.

Upon transport to the laboratory, the sediment cores were divided into three layers (0–20, 20–40, 40–65 cm) (Fig. 2). The cliff and sea sediments were transferred into polyethylene bags and frozen at − 20 °C (holding time 48 h). The samples were then lyophilised (Alpha 1-4 LDplus, Martin Christ) and homogenised in a ball mill (8000D, Mixer/Mill, SPEX) with a tungsten vessel. Part of the material was additionally analysed for basic sediment parameters: organic matter content, granulometry and water content.

### Laboratory analysis

The analyses were carried out maximum 30 days after lyophilisation. The analyses were performed using a clean environment and non-metallic labware to avoid sample contamination, according to US EPA 1631 method (US EPA 2002). Hg analyses were performed on a direct mercury analyser DMA-80 (Milestone). Hg speciation was determined using the thermodesorption method described by Saniewska and Bełdowska (2017), modified by Bełdowska et al. (2018) and Jędruch et al. (2018a). The samples were weighted (sample mass 0.1 g) on previously digested in 3–4 M HNO_3_ and ignited (800 °C, 1 min) quartz glass sample boats. The sediment samples were successively incinerated at the following temperatures: 175, 225, 325, 475 and 750 °C, in order to distinguish between labile (bioavailable) and stable forms of Hg and to identify their proportions in the total Hg (Hg_TOT_). The first fraction consists of loosely bound Hg compounds, released at the lowest temperature of 175 °C (HgCl_2_, HgBr_2_, HgI_2_, Hg(CN)_2_), mainly adsorbed on the surface of sediment particles (Hg_ads1_) (Bełdowska et al. 2018). At a temperature of 225 °C, labile hummus-like substances are released, as well as MeHg (monomethyl mercury) (Saniewska and Bełdowska 2017)—the most toxic for organisms and compounds absorbed in organic matter (Hg(SCN)_2_, (CH_3_COO)_2_Hg, Hg(NO_3_)_2_), Hg(ClO_4_) (Hg_abs_)) (Molina et al. 2010). 325 °C triggered the decomposition of HgS, one of the most stable Hg forms. At 475 °C, the released forms were mainly adsorbed HgO, HgSO_4_ and HgF2 (Hg_ads2_), potentially available to organisms (Sadiq 1992). The Hg compounds that decomposed at the highest temperature of 750 °C were in a form unavailable to the environment bounded to the residual fraction incorporated in mineral matrix (Hg_res_) (Bełdowska et al. 2018). The method quality was verified by the analysis of certified reference materials differing in terms of Hg_TOT_ level and organic matter content (tea leaves INCT-TL-1—Hg_TOT_ 5 ng g^−1^, soil NCS DC 87103—Hg_TOT_ 17 ng g^−1^, plankton BCR-414—Hg_TOT_ 276 ng g^−1^, marine sediment GBW 07314—Hg_TOT_ 48 ng g^−1^), used in previous research conducted by the authors (Jędruch et al. 2017; Saniewska and Bełdowska 2017; Bełdowska et al. 2018; Jędruch et al. 2018a). The CRM’s analyses were carried out in three replications, for which the average recovery was respectively 105, 96, 104 and 98%. The limit of detection (LOD), calculated as the threshold of the standard deviation of Hg concentration in the blank samples, for each fractionation method, was calculated from ten replicates of the substrate analysis. The calculated LOD values were at the level of 1 pg of Hg.

The content of water in collected sediments was determined by drying the sample at 60 °C for 24 h (Winters 2000). The organic matter content, expressed as loss on ignition (LOI) (Santisteban et al. 2004), was determined by heating sediment samples at 550 °C for 6 h, which is described as the best method for Baltic sediments (Ciborowski 2010). Granulometry, using sieve analysis, was carried out to determine the proportion of individual sediment fractions in the studied samples. The sediments were sieved using a mechanical shaker for 10 min, through the following mesh sizes: 2, 1, 0.5, 0.25, 0.125 and 0.063 mm. The sizes of individual fractions were determined using the Udden classification (Udden 1914) modified by Wentworth (1922). The sediments with a diameter below the 0.063 mm were defined as a fine-particle sediment fraction (FSF).

The Hg loads to the Gulf of Gdansk as a result of cliff erosion were estimated in accordance with the method described in previous studies by the authors, conducted in 2011–2014 (Bełdowska et al. 2016). For this purpose, apart from Hg concentration in the abraded sediment material, changes in the active surface of the cliff walls over time were also analysed using aerial laser scanning (airborne LiDAR) of selected coast sections—the detailed data can be found in work by Bełdowska et al. (2016).

### Processing of the results

The presented concentrations of Hg_TOT_ and Hg fractions in the collected material were expressed in terms of dry weight (dw).

Statistical analysis and graphic representation of the obtained results were carried out using *STATISTICA 12* software (StatSoft). The normality of data distribution was analysed using Shapiro-Wilk test (*p* < 0.05). In order to determine the significance of differences, the non-parametric Mann-Whitney *U* or Kruskal-Wallis tests were used (*p* < 0.05). The relationships between the analysed variables were determined on the basis of the Spearman’s coefficient, with a confidence interval of at least 95%.

The map of the study area with the distribution of sampling stations was created using *ArcGIS 10.4* (ESRI) software with the geographic coordinate system chosen for data presentation WGS1984. The spatial data were provided courtesy of the GIS Centre, University of Gdańsk (www.ocean.ug.edu.pl/~oceju/CentrumGIS).

## Results and discussion

### Inflow of labile mercury with the erosion of the cliff colluvium

#### Labile mercury in the cliff colluvium

In all the analysed cliff sediment samples taken from the surface layer (0–20 cm) of the colluvium, the Hg_TOT_ concentration ranged from 1.5 to 13.9 ng g^−1^ (Table 1a). These results were similar to those obtained in the studies of cliff sediments carried out in that region in previous years (Bełdowska et al. 2016). Hg_TOT_ concentrations in the cliff material (mean 9.7 ng g^−1^) did not exceed the value considered to be the natural geochemical Hg background in the Baltic Sea region, amounting to 20–30 ng g^−1^ (Korhonen et al. 2001; Liepe et al. 2013). They were also several times lower than the Hg_TOT_ level measured in the surface sediments of the open part of the Baltic Sea (Bełdowski et al. 2014; Jędruch et al. 2015). However, the values measured in the cliffs were several times higher than the Hg_TOT_ concentrations measured in the surface sediments of the Gulf of Gdansk coastal zone (mean 1.8 ng g^−1^) (Jędruch et al. 2018b). The highest concentrations of Hg_TOT_ were measured in sediments collected at the Mechelinki cliff (range 8.4–13.9 ng g^−1^), slightly lower ones were measured in the Orlowo (range 9.6–12.8 ng g^−1^) and Oslonino cliff sediments (range 8.1–12.3 ng g^−1^), while the lowest were those in the Puck cliff sediments (range 3.7–5.6 ng g^−1^) (median concentrations are given in Table 1). The differences in metal concentrations in the studied cliffs were associated primarily with their geological structure and thus with sediment type: the domination of sands, gravel or clay sediments in the particular cliffs, as well as with the content of organic matter (Table SI). It is crucial because Hg is characterised by high affinity to a fine fraction (< 0.063 mm) of sediments (Pempkowiak 1997). Taking into account the results from the all studied cliffs, a statistically significant difference in Hg_TOT_ content was observed between two sediment types: boulder clay—the main building material of the cliffs in the Gulf of Gdansk region—and sandy sediment layers (Mann-Whitney *U* test, *p* = 0.00). Hg_TOT_ concentrations in sediments collected from the colluvium (horizontal cores) were also positively correlated with the sediment parameters: the content of the fine fraction (*R* Spearman = 0.70) and of organic matter (LOI) (*R* Spearman = 0.60) (Table SIII). This explains the fact that the Hg_TOT_ concentrations measured in the fine-grained clay sediments were about twice as high as in the coarser sandy sediments. It also explains the lowest Hg_TOT_ values in the Puck cliff sediments, where the proportion of the fine particle sediment fraction was the lowest (Table SI), and the occurrence of coarse-grained sediments (sands and gravel) was the highest. The different compositions of the Puck cliff are also confirmed by the results for concentrations of other metals (Ti, V, Cr, Mn, Fe, Cu, Zn, Rb, Zr), measured in parallel studies conducted by the authors (Kwasigroch et al. 2016), which were statistically significant different (Kruskal-Wallis test, *p* < 0.05) compared to the results obtained for other cliffs.

**Table 1:** Total Hg (Hg_TOT_) concentration and labile Hg percentage (medians and range) in (a) horizontal and (b) vertical profiles of investigated cliffs

		Layer (cm)		
		0–20	20–40	40–65
a)				
Orłowo	Hg_TOT_ (ng g^−1^)	10.2 (9.6–12.8)	–	–
	Hg_labile_ (%)	64.1 (59.2–68.4)	–	–
Mechelinki	Hg_TOT_ (ng g^−1^)	13.3 (11.8–13.9)	8.9 (8.3–9.2)	8.4 (8.1–8.5)
	Hg_labile_ (%)	74.2 (72.6–76.0)	76.3 (72.1–77.8)	72.2 (70.8–74.1)
Osłonino	Hg_TOT_ (ng g^−1^)	10.5 (10.3–10.6)	11.9 (10.6–12.3)	8.3 (8.1–8.6)
	Hg_labile_ (%)	63.2 (55.0–64.9)	65.2 (58.1–67.3)	60.6 (56.3–63.6)
Puck	Hg_TOT_ (ng g^−1^)	4.7 (3.7–5.6)	5.1 (4.7–5.5)	4.8 (4.4–5.4)
	Hg_labile_ (%)	60.9 (58.2–69.4)	64.1 (59.4–66.3)	63.4 (58.2–65.2)
b)				
Orłowo	Hg_TOT_ (ng g^−1^)	26.9 (18.8–34.5)	–	–
	Hg_labile_ (%)	86.3 (74.1–91.0)	–	–
Mechelinki	Hg_TOT_ (ng g^−1^)	25.4 (20.2–29.3)	18.0 (13.1–32.0)	19.5 (15.2–24.5)
	Hg_labile_ (%)	71.4 (63.4–79.0)	53.3 (39.1–69.1)	57.1 (45.9–60.3)
Osłonino	Hg_TOT_ (ng g^−1^)	28.9 (26.0–32.1)	17.1 (15.9–18.1)	19.4 (14.4–24.0)
	Hg_labile_ (%)	63.5 (57.6–64.2)	53.6 (49.1–58.1)	48.1 (44.3–51.2)
Puck	Hg_TOT_ (ng g^−1^)	21.6 (18.6–24.7)	13.4 (8.7–19.4)	7.9 (5.7–10.1)
	Hg_labile_ (%)	63.2 (58.0–66.5)	63.5 (58.6–68.8)	60.1 (58.2–62.1)

**Fig. 3:**
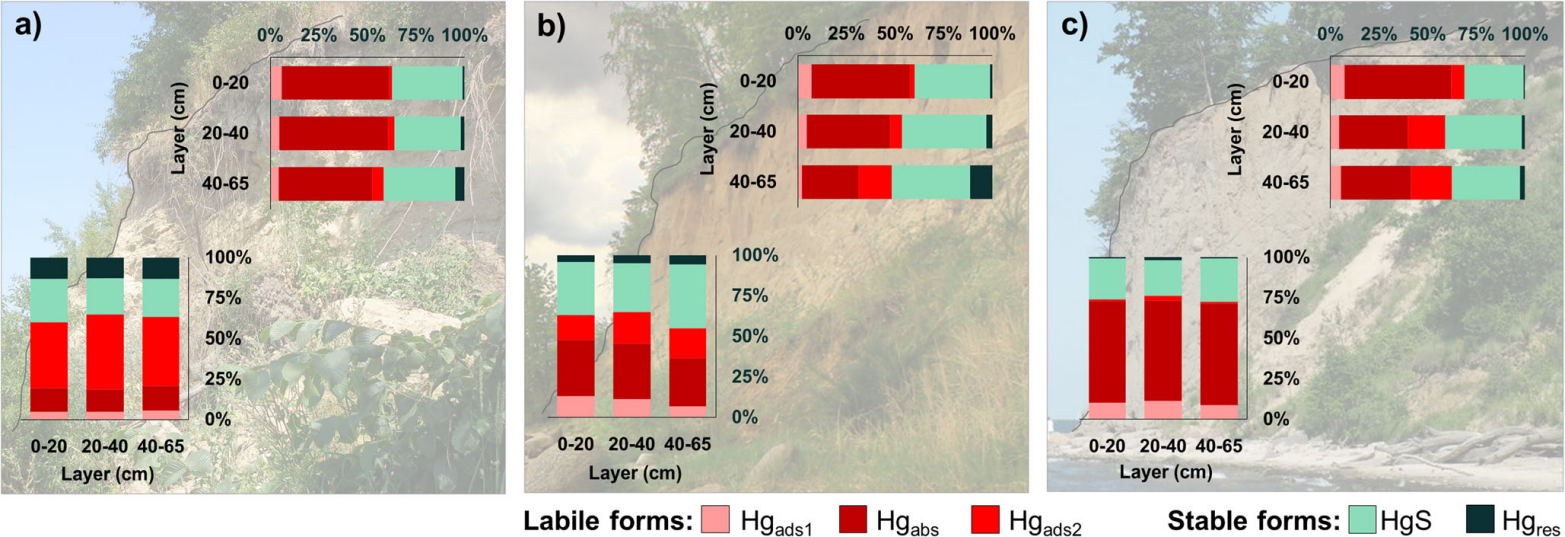
The percentage of Hg fractions in horizontal and vertical profiles of investigated cliffs. **a** Puck cliff. **b** Oslonino cliff. **c** Mechelinki cliff

The share of labile Hg (Hg_labile_) forms in Hg_TOT_ ranged from 50.3 to 76.0% (Fig. 3; Table 1a). As was the case with Hg_TOT_ concentrations, the highest percentage of Hg_labile_ was measured in the Mechelinki cliff sediments (74.2%; Hg_labile_ median 9.9 ng g^−1^), the second highest in the Orlowo cliff (64.1%; Hg_labile_ median 6.5 ng g^−1^), then the Puck cliff (60.9%; Hg_labile_ median 2.8 ng g^−1^) and finally the Oslonino cliff (57.0%; Hg_labile_ median 6.6 ng g^−1^). There was no statistically significant difference between the concentrations of labile Hg observed in clay sediments and those found in sandy cliff sediments (Mann-Whitney *U* test, *p* > 0.05).

#### Labile mercury load introduced with erosion of the cliff colluvium

Owing to the geological structure of the cliffs on the Polish coast, a significant part of which (about 80%) consists of boulder clay formations (Subotowicz 1980); only the values for this sediment type (0–20 cm layer of horizontal cores) were taken into account when calculating the Hg_TOT_ loads reaching the marine environment with the erosion of the cliff colluvium. The average concentration of Hg_TOT_ in the boulder clay of the studied cliffs was 9.7 ng g^−1^. Bearing in mind the volume and mass of sediments that crumble into the Gdansk Basin annually (362–672 t) (Bełdowska et al. 2016), the calculated Hg_TOT_ load amounted to a total of 15.6 kg a^−1^. This value is comparable to that estimates by the authors for data from years 2011–2014 (14.3 kg a^−1^), and a slight difference was caused by lower Hg_TOT_ concentration in boulder clays (mean 8.8 ng g^−1^) measured in previous years (Bełdowska et al. 2016). The calculated Hg_TOT_ load introduced to the Gdansk Basin as a result of coastal abrasion was almost as high (87%) as the load of Hg_TOT_ reaching the basin along with the precipitation (17.9 kg a^−1^) and over 60% higher than the dry atmospheric deposition (9.5 kg a^−1^) of Hg_TOT_ (Bełdowska et al. 2014, 2016). What is important, the Hg_TOT_ load introduced to the Gdansk Basin with abraded sediments was also over five times greater than the combined inflow of Hg_TOT_ (2.7 kg a^−1^) via small rivers (Reda, Zagorska Struga, Plutnica and Gizdepka), with an average water flow from 0.2 to 4.2 m^3^ s^−1^, into the study area (Saniewska et al. 2018).

In the boulder clay sediments of all the analysed cliffs, the percentage of labile Hg in Hg_TOT_ was over 50%. The calculated shares were slightly lower than the values (ca. 70% of Hg_labile_) recorded in soils with low Hg_TOT_ level (ca. 20 ng g^−1^) and low organic matter content (ca. 5%) (Saniewska and Bełdowska 2017). However, it is important that despite the percentage of LOI in these soils was low (for a usually organic-rich matrices like soils), it was still about two times higher than organic matter content measured in cliff sediments (Table SI).

Taking into account the average labile Hg fraction content (64%) in individual cliffs, it was estimated that its load in the sediment material that is introduced annually to the Gdansk Basin amounts to 10.0 kg a^−1^. It is worth stressing here that the load of labile Hg consists mainly of Hg absorbed in organic matter (Hg_abs_). An exception was the Puck cliff, where Hg predominated in the form of HgO and HgSO_4_ (Hg_ads2_) (Fig. 3). Probable explanation for such a large proportion of Hg_ads2_ (41–46%) in the colluvial sediments collected from the Puck cliff is the occurrence of brow coal complexes (Lower and Middle Miocene lignite) in the walls of this cliff, as well as in other cliffs located in the northern part of the Puck Bay and in the area to the west of the Hel Peninsula (Wagner 2007). Similar results were also presented in the study by Bombach et al. (1994), in which the samples rich in brown coal showed a different behaviour of Hg release than other natural samples (soils, sediments)—as a result of thermal prehistory, the most of Hg was released in temperatures above the 400 °C (410–480 °C), which corresponds to the temperature (475 °C) at which Hg_ads2_ decomposed.

### Inflow of labile mercury with the erosion of the cliff top

During strong winds and heavy storms, the upper parts of the cliffs also undergo erosion. At such times, a part of the upper cliff wall can detach itself or some of the sediment material can slide from the top. The cliffs of the gulf are mostly overgrown with trees and other vegetation, and some of them are in the vicinity of arable land, which has a significant impact on soil and cliff deposits properties (Doody 2001; Neris et al. 2012) and, consequently, on the Hg content (Lacerda et al. 2004). In order to investigate the impact of cliff top erosion on the inflow of labile Hg_TOT_ into the gulf, vertical sediment cores (0–65 cm) were collected from the tops of the studied cliffs.

The Hg_TOT_ concentrations in the surface layer (0–20 cm) of the sediment taken from the cliff tops were significantly higher than the values measured in the sediments collected from the base of the cliff. In the case of the samples collected from the tops of three analysed cliffs—the Orlowo cliff (median 29.6 ng g^−1^), the Oslonino cliff (median 28.9 ng g^−1^) and the Mechelinki cliff (median 25.4 ng g^−1^), the measured values were two times higher than in the surface layer of the colluvia of these cliffs, whereas in the case of the Puck cliff (median 21.6 ng g^−1^), the value was four times higher (Table 1b). The obtained values are more characteristic for soils than for cliff sediments (Jaworska et al. 2009; Falandysz et al. 2012; Bełdowska et al. 2016; Różański et al. 2016). This is due to the fact that the cliff tops are densely overgrown with vegetation, which enriches the top soil with organic matter of strong Hg complexing properties. The high share of organic matter in sediments collected from the cliff top surface is confirmed by the high LOI values (Table SI). The high content of organic matter in the 0–20 cm sediments also resulted in an increased share of labile Hg, especially in the case of the Orlowo cliff (86.3%), which top is characterised by an exceptionally rich vegetation, compared to the other cliffs. The share of labile Hg was slightly lower at the Mechelinki cliff (71.4%), while the smallest content of this Hg fraction were recorded at the Osłonino (63.5%) and Puck (63.2%) cliffs (Fig. 3; Table 1b). The share of organic matter was an important factor determining the level of Hg_TOT_ in the vertical cliff profile—a statistically significant, strong positive correlation between LOI and Hg_TOT_ concentrations in sediments was demonstrated (*R* Spearman = 0.79). In addition, as was the case with horizontal cores, the Hg_TOT_ concentrations in vertical core sediments increased along with the proportion of fine-particle sediment fraction (*R* Spearman = 0.88) (Table SIII). As the share of organic matter and fine-particle sediment decreased deeper into the collected vertical cores, the Hg_TOT_ concentrations also decreased (Table SI). This trend was also reported by other researchers in the case of riverside soils (Bombach et al. 1994), as well as in the case of forest and arable soils (Pokharel and Obrist 2011; Różański et al. 2016). Except to the affinity of Hg to organic matter and fine sediment fraction, the changes of the Hg_TOT_ concentration in the sediments profile can be related to the water content affecting the Hg sorption (Pokharel and Obrist 2011)—the highest wetness of sediments was measured in the surface layers (0–20 cm) (Table SI).

In the case of the Osłonino cliff, the Hg_TOT_ concentrations in the 20–40 cm layer (median 17.1 ng g^−1^) and in the 40–65 cm layer (median 19.4 ng g^−1^ dw) were about 40% lower than in the surface layer (median 28.9 ng g^−1^) (Table 1b). Also, the share of labile Hg was smaller in the lower layers than in surface sediments—in the 20–40 cm layer (53.3%), it was about 15% lower, and in the 40–65 cm layer (48.1%), it was 25% lower (Table 1b). The greatest share of Hg_labile_ in the surface layer resulted from the high content of the organic matter, as well as humic and fulvic substances originated from the decomposition of the vegetation covering the cliff top (Shindo 1991). The enrichment of the topmost sediments in organic compounds was reflected in a significant share of the Hg_abs_ form (51.3% of total Hg) (Fig. 3). The Hg speciation in this layer was also associated with the sorption of atmospheric Hg (Pokharel and Obrist 2011)—the percentage of the fraction decomposing at lowest temperatures (Hg_ads1_) was the highest in the entire profile (7.1%) and over three times greater than in the deepest layer (2.2%) (Fig. 3). This is confirmed by the results of previous studies by authors (Bełdowska et al. 2018), which showed that atmospheric Hg (gaseous elemental and reactive Hg, Hg halides adsorbed in the aerosol) was released at similar (or lower) temperatures as Hg_ads1_ (125–175 °C). The distribution of the Hg speciation in the collected sediments also indicates an increase in stable Hg compounds, especially those bound with sulphides (Fig. 3), which is related to the fact that the deeper mineral layers of the core provide a sink for stable, non-mobile Hg (Pokharel and Obrist 2011). In the vertical core taken from the Mechelinki cliff, the Hg_TOT_ concentrations in the 20–40 cm layer (median 18.0 ng g^−1^) and the 40–65 cm layer (median 19.5 ng g^−1^) were similar, as in the case of deeper sediment layers from the Osłonino cliff. However, the difference between the levels of Hg_TOT_ in these layers and in the surface layer was as high as 30%. The lowest share of labile Hg in the vertical profile in Mechelinki was observed in the 20–40 cm layer (53.3%)—this value was about 25% lower than in the surface sediment layer. In the deepest layer of 40–65 cm, the share of labile Hg was slightly higher (57.1%) and 20% lower than in the 0–20 cm layer. In the Puck cliff, the variability of Hg_TOT_ concentrations in the vertical profile was the highest compared to the other cliffs—the level of metal concentrations in the 20–40 layer (median 13.4 ng g^−1^) was 40% lower than in the surface layer, while in the lowest level (median 7.9 ng g^−1^) up to 65% lower (Fig. 3). The Puck cliff differs from the other studied cliffs in terms of composition: in addition to boulder clay, a significant part of it is formed of sand and gravel sediments (Subotowicz 1982; Wagner 2007), as confirmed by granulometric analyses showing that the proportion of sediment with larger grain diameter (> 0.25 mm) is almost two times higher than in the other cliffs. This has influenced the lower concentrations of Hg in the deeper layers of the cores. Interestingly, in the case of the Puck cliff, the variability along the core was not observed for the labile Hg fraction—in the case of both the 20–40 cm layer (63.5%) and the 40–65 cm layer (60.1%), the obtained values were similar to the surface layer (Fig. 3).

#### Hg load during the abrasion of the cliff wall

Owing to the climate change, the effects of which are already visible, in the Southern Baltic region, we observe, among other things, an intensified occurrence of extreme meteorological phenomena such as strong storms and heavy rains (HELCOM 2013), which may lead to the abrasion of larger, upper parts of cliff walls. It is therefore important to include this fact in the calculated Hg loads into the gulf. Here, to estimate the load, the mean concentration of labile Hg in the colluvium was taken into account, as well as the concentration measured in the entire cross section of the vertical core collected from the cliff top. This enabled to assess the increase in Hg load in a case when erosion is not only of the cliff colluvium but also of a part of its wall.

When calculating the load according to the above assumption, there was an almost 50% increase in the labile Hg load to the Gulf of Gdansk (14.9 kg year^−1^). However, the average share (in the surface layer of the colluvium and cores from the cliff top) of labile Hg was lower than for the percentage share of labile Hg in the colluvium alone (Table 1a, b). This is due to the fact that in deeper cliff layers, with no direct influence of atmospheric factors, rainwater infiltration, etc., Hg is more commonly found in stable bonds, e.g. with sulphides (Sadiq 1992) (Fig. 3).

### The forecast inflow of labile Hg with cliff erosion

In order to estimate the future Hg load introduced into the Gulf of Gdansk via the erosion of cliffs, horizontal cores were collected from the walls of the investigated cliffs (Fig. 2). The surface layer of the core (0–20 cm) was a part of the colluvium, for which current Hg loads from particular cliffs into the gulf were calculated. The deeper layers, 20–40 and 40–65 cm, were analysed in order to assess the change in Hg_TOT_ concentrations and the share of the labile Hg fraction along the horizontal sediment profile. As was the case with sediments from the surface layer, a statistically significant difference in Hg_TOT_ concentrations was found between sandy sediments and boulder clay (Mann-Whitney *U* test, *p* = 0.00). However, in contrast to vertical cores, no statistically significant difference was found between the Hg_TOT_ concentration and the share of individual labile and stable fractions in the surface layer (0–20 cm) of cliffs and in deeper layers of the core (Mann-Whitney *U* test, *p* = 0.00). This suggests that the cliff colluvial sediment is well mixed (down to a depth of at least 65 cm) and significant differences in concentrations would most likely be noticed only when collecting considerably longer cores of several meters, which would give a chance of reaching intact sediment. This is confirmed by the findings published by Bombach et al. (1994) or Różański et al. (2016), indicating that significant changes in the composition (i.e. concentration of Fe and Mn) and properties (i.e. pH) of sediments being an important factors controlling the level of Hg (Pempkowiak 1997), compared to the surface layer, may appear at the depth of about 80 cm or more. As a consequence, also the important changes in the Hg concentration were observed at similar depths (greater than the length of the cores collected by the authors of this study).

Due to lack of the variability of Hg concentration with the depth of the sediments collected from the cliff walls, it can be concluded that the inflow of Hg into the gulf is not going to change in the coming years, and any possible increase of inflow can be caused by rapid erosion of the cliff top as a consequence of extreme weather phenomena whose frequency in the study area increases (Kożuchowski 2009).

### Transportation of mercury from cliff erosion into the Gdansk Basin

**Fig. 4:**
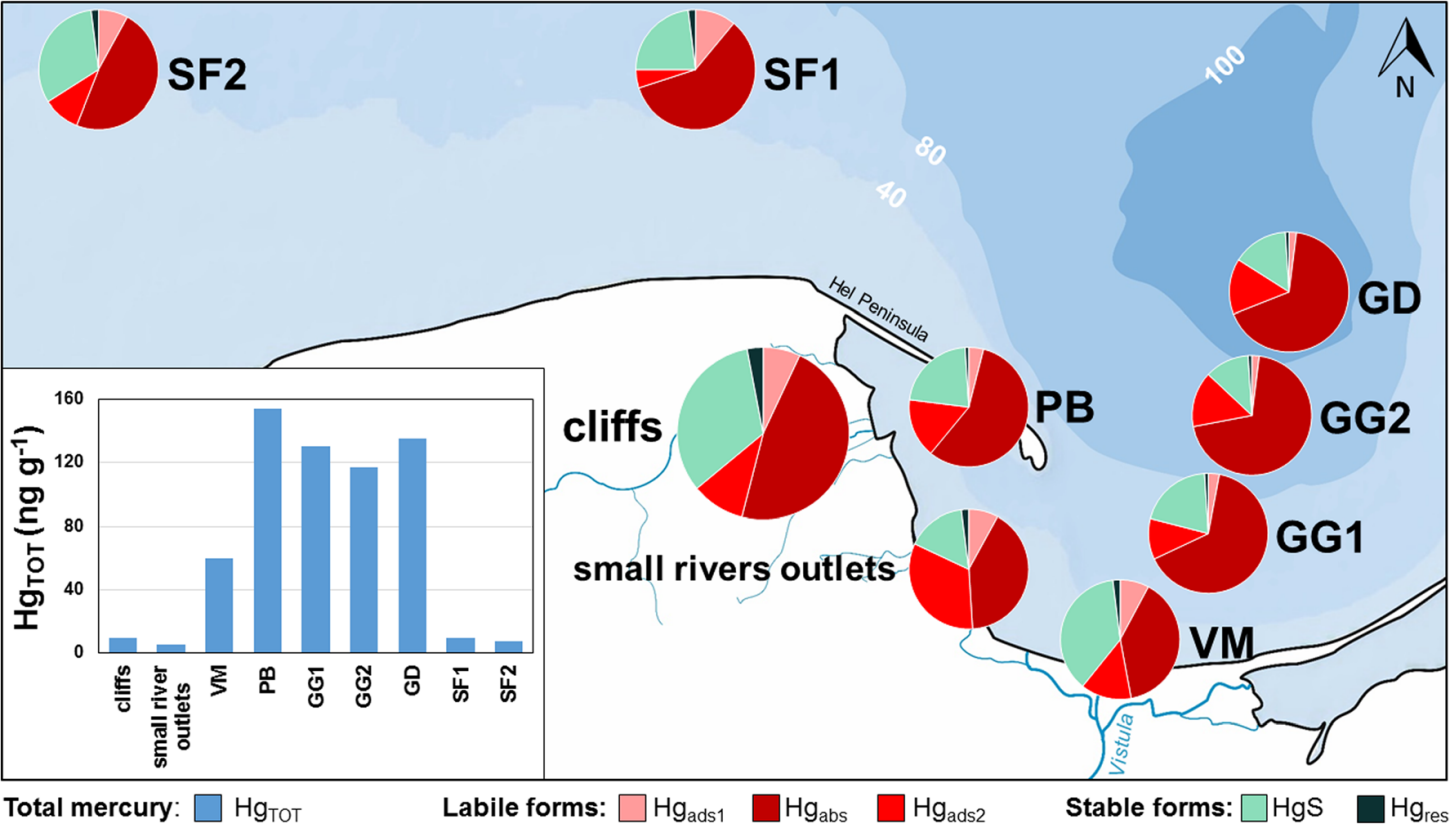
The total Hg (Hg_TOT_) concentration and the percentage of Hg fractions in sediments collected from the cliffs and from the different types of sea bottom

In order to estimate the potential sedimentation area of the Hg originating from the cliff coast erosion, the total Hg content and the share of individual Hg fractions in the cliff sediments and in the three sea bottom types (areas of erosion, transportation and accumulation) were assessed (Fig. 4; Tables SI and SII).

In all of the studied cliff sediments (Fig. 1), labile Hg fractions (Hg_ads1_, Hg_abs_, Hg_ads2_) were predominant. Their combined share was 64% on average. The largest (47%) average share was estimated for the Hg absorbed in organic matter (Hg_abs_). In the landslide material (horizontal cores 0–65 cm) of the Mechelinki cliff, that share was the highest and amounted to an average of 63%. This was related to the fact that the material at that station was characterised by the highest content of organic matter among the studied cliffs (Table SI). The content of organic matter was lower by more than a half in the case of the other cliffs. For this reason, in those cliffs, the share of Hg_abs_ was almost half as low but still predominant. An exception was the Puck cliff, where the dominant Hg fraction (43%) was Hg bound with oxide and sulphate. That fraction negatively correlated with the content of organic matter in the cliff sediment (*R* Spearman = − 0.47). In the remaining cliffs, the average content of this fraction in the colluvial material was more than four times lower and amounted to 10%.

Among the labile fractions, the lowest share was that of Hg bound with halides, adsorbed (Hg_ads1_) on the surface of organic matter or the fine sediment fraction. The average share of this fraction was 7% and was very similar in all the horizontal and vertical profiles in the examined cliffs. As far as stable fractions are concerned, the Puck cliff differed from all the other cliffs in terms of the content of the stable, residual Hg. The average content of not bioavailable, residual Hg in the remaining cliffs was 3%, while in the Puck cliff, that value was more than four times higher and amounted to 13%. In the cliff landslide material, the content of this fraction did not change with depth due to good sediment mixing. Another stable Hg fraction, which was possible to distinguish in the sediments, was Hg sulphide (HgS). Hg forms stable complexes with sulphur, which can transform into more bioavailable forms only as a result of changes in oxy-reduction conditions (Bełdowski and Pempkowiak 2009). The content of this fraction was similar in all the cliffs and amounted to an average of 33%. In contrast to residual Hg, there were no significant changes in HgS content in either vertical or horizontal core.

In sediments collected from beaches in the cliff areas and from the shallow coastal zone (stations were placed analogously to those of the particular four cliffs), the dominant Hg fraction was Hg_abs_ (Table SI). In beach sand, its share amounted to 47%, while in sea sediment, it was 44%. However, due to the fact that the Hg_TOT_ concentration median was very low in both cases (beach sand median = 1.1 ng g^−1^, coastal sediment median = 1.8 ng g^−1^), it is not possible to make conclusions about the accumulation of sedimentary material from cliffs in their close vicinity. On the beach and in the shallow coastal zone, the sediments are coarse (fine fraction median in beach sand = 0.03%, in coastal sediment = 0.2%) and contain a minuscule amount of organic matter (LOI median in beach sand = 0.4% and in coastal sediment = 0.9%) (Table SII). The average share of the Hg fraction bound with oxides and sulphates was the same in both cases and amounted to 9%. The difference was in the case of stable Hg fractions. Residual Hg predominated in beach sand (17%) compared to HgS (15%), while in the shallow coastal zone HgS (25%) markedly dominated over the most stable fraction: residual Hg (5%).

In sediments collected from the estuaries of four rivers near the studied cliffs (Fig. 1a), labile Hg was predominant, as in the cliff sediments (Fig. 4). However, the proportions of individual Hg fractions were slightly different. The highest average share (41%), similar to the values obtained for cliff sediments, was that of Hg absorbed in the organic matter, but equally important (33%) was the share of the Hg fraction bound with oxides and sulphates (Table SII), which suggests good oxygenation of waters in the estuary, hindering the formation of the reduced Hg form (Bełdowski and Pempkowiak 2009). This is also influenced by the fact that the dominant form of Hg in water in small rivers is the dissolved Hg (Saniewska et al. 2014). This is evidenced by the low Hg sulphide content (HgS), at a level of 12% in river estuary sediments, which is more than two times lower than in cliff sediments. The share of residual Hg was small and did not exceed 2%.

Samples from the marine stations (Fig. 1b) were collected in three regions representing different seabed types: transportation seabed in the Hel Peninsula region (station PB) of the Gulf of Gdansk and the Slupsk Furrow (stations SF1 and SF2), temporary accumulation seabed in the central Gulf of Gdansk (stations GG1 and GG2), accumulation seabed in the Gdansk Deep area (station GD) and in the area of the erosive seabed by the River Vistula outlet (station VM) (Staśkiewicz 2009; Uścinowicz 2009) (Fig. 1b). At all stations, the dominant Hg fraction was Hg absorbed in organic matter, which was closely related to the organic matter content (*R* Spearman = 0.71) (Fig. 4). It is in agreement with previous studies in the area, where Hg bound to humic and fulvic acids dominated in the Gulf of Gdańsk and Gdańsk Deep (Bełdowski and Pempkowiak 2009).

In the area of the erosive seabed, in the estuary of the River Vistula (St. VM) (median Hg_TOT_ 59.8 ng g^−1^), the second largest river entering the Baltic Sea in terms of the catchment area, the shares of the individual fractions were more similar to cliff sediments than to sediments from the small river estuaries (Fig. 4). The prevalent fraction was that of Hg absorbed in organic matter, constituting 40% of Hg_TOT_. On the other hand, the fraction of Hg bound with sulphates and oxides was more than a half lower than in small rivers, amounting to 14%. However, the stable HgS fraction (36%) was more than twice as large. This was related to the fact that the dominant form of Hg in the water of the River Vistula is suspended Hg (ca. 80%), as opposed to small rivers (Saniewska et al. 2014).

In the Hel Peninsula region (St. PB) (median Hg_TOT_ 153.8 ng g^−1^) (Fig. 1a, b), at the marine station located the closest to the analysed cliffs, a high share of Hg_abs_ (57%) was found, but the content of organic matter was lower than in the area of the accumulation seabed (LOI = 8.6%) (Table SII). This indicates that the organic matter present in sediment in that region was more enriched with Hg. The Hg fraction bound with oxides and sulphates was at a similar level to the other marine stations and amounted to 16%. Among the stable Hg fractions, residual Hg accounted for a very small share (1%), while HgS constituted 22%, which was almost twice as high as in the accumulation bottom (Fig. 4). This is related to the shape of the seabed in the sample collection area. The seabed in the area of the station is significantly deeper (35 m) than the surrounding bottom (the average depth of Puck Bay is approx. 3 m).

In the Slupsk Furrow region (St. SF1, St. SF2) (median Hg_TOT_ 9.7 and 7.1 ng g^−1^, respectively) (Fig. 1b), the share of Hg_abs_ (53%) was very similar to that obtained for cliff sediments and that was associated with a lower organic matter content than in the accumulative seabed area. The LOI values obtained in this region were more similar to those of the cliff colluvium, where the content of organic matter did not exceed 2–3% (Tables SI and SII). The share of the stable HgS fraction was similar to that obtained for cliff sediment and amounted to 28% (Fig. 4). Due to the transportive nature of the seabed and sandy sediment quality, the concentrations observed here were relatively low. However, taking into account the distribution of the shares of individual fractions, it can be concluded that the sediment located there may originate from the coastal erosion. The highest share of Hg_abs_, which was almost 50% higher than in cliff sediments, was found at the stations in the Gdansk Deep (St. GD) (median Hg_TOT_ 135.6 ng g^−1^) and central Gulf of Gdansk (St. GG1, St. GG2) (median Hg_TOT_ 130.5 and 116.9 ng g^−1^, respectively) and amounted to an average of 69%, including the highest organic matter content (up to 14%) (Table SII). Adsorbed Hg constituted a small share (3%), and the Hg fraction bound with sulphates and oxides accounted for 14%. This indicates that the content of organic matter determined the shares of individual labile Hg fractions. The share of stable fractions was definitely lower. The residual Hg content was marginal (1%), and the HgS share was at a level of 13%, which was almost three times lower than that obtained for cliff sediments.

From the above analyses, it can be concluded that, based on the percentage distribution of individual Hg fractions in sediments from accumulative regions, it is not possible to isolate the “cliff signal” in them. The cliff sediments are not “retained” in the Gulf of Gdańsk due to the high content of fine sediment fraction. Instead, they are transported over longer distances and do not accumulate in the coastal zone area. Transport of fine particulates from the coastal zone into remote accumulation areas was previously reported in the Baltic Sea, in the form of unconsolidated sediments travelling via subsequent resuspension-deposition cycles (Pempkowiak et al. 2002, 2005; Uścinowicz 2011). However, the number of processes that can affect the percentage of particular Hg fractions in deeper regions, where sediments accumulate, is so high (i.e. adsorption and formation of chemical complexes, changes of redox conditions, biotic and abiotic Hg methylation and demethylation, resuspension and diffusion) (Sadiq 1992; Zhang and Planas 1994; Jackson 1998; Bełdowski and Pempkowiak 2009) that the “print” of cliff sediments is wiped away. The transportation of large amounts of suspension via the Vistula River is of great importance here, as well as processes in situ, conditioned by, e.g. high organic matter content, oxygen conditions and inflows of water from the North Sea.

## Conclusion

The concentrations of Hg_TOT_ in cliff sediments from the horizontal cores collected from the cliff colluvia did not change alongside the core, indicating the homogeneity of the sediment material there. In the case of the vertical cores, collected from the top of the cliff, a decrease in Hg_TOT_ concentration was observed with depth, which was related to the high content of organic matter in the surface layer of the collected cores. The average content of labile forms in the cliff sediments was 64%. In the surface layer of the vertical cores, taken from cliff tops, a higher share of labile Hg fractions was found than in the horizontal cores from cliff colluvia and that was associated with the higher organic matter content mentioned above. In contrast, in the deeper layers of the vertical cores, the share of labile Hg dropped below 50%. For all of the studied cliffs (except the Puck cliff), the predominant fraction of labile Hg was Hg_abs_. A situation analogous to that in the distribution of Hg_TOT_ concentrations was observed in the share of individual Hg fractions. In the horizontal cores, the shares of individual Hg fractions were very similar throughout the core cross section. In contrast, the vertical cores showed a decrease in the fraction of Hg absorbed in organic matter, deeper into the core. This was accompanied by an increase in stable fractions, especially residual Hg.

After taking into account the average share of labile Hg fractions in cliff sediments and in Hg_TOT_, it was estimated that the annual load of labile Hg into the Gdansk Basin is 10.0 kg a^−1^. Assuming the accuracy of climate change forecasts (HELCOM 2013), which predict an increase in storm and rainfall intensity; in extreme cases, the erosion of entire cliff faces may be expected. In that case, the load of labile Hg into the Gdansk Basin may increase by almost 50%, up to 14.9 kg a^−1^.

Despite the fact that Hg_TOT_ concentrations in cliff sediments are at the level of the geochemical background, taking into account the large mass of sediments that enter the Gdansk Basin each year, it should be emphasised that Hg from coastal erosion is an important source of Hg into the marine environment. Hg from cliff erosion is not deposited in the beach sand nor in the coastal zone, but it is transported to deeper regions of accumulation, located at a relatively long distance from the shore (and consequently from the land-based Hg sources). Although the exact contribution of the “cliff print” in marine sediments could not be determined, cliff sediments can affect the bioavailability and mobility of Hg in the open sea bottom. As a consequence, the Hg level, as well as the share of bioaccessible forms of Hg in marine sediments, can increase.

The high content of labile fraction in sediments collected from the cliffs suggests that Hg from this source can be easily included in the marine trophic chain, as confirmed by previous studies (Bełdowska 2016; Bełdowska et al. 2016). It should be noted that Hg from this source is introduced over several episodes per year, which exposes organisms living in the coastal zone to increased doses of bioavailable Hg in a short time period. The significant amounts of labile Hg can be easily introduced to the trophic chain as a result of remobilisation from marine sediments as well. This means that marine sediments should not be considered as a Hg sink, but as a place of its temporary accumulation.

## Electronic supplementary material

ESM 1 (PDF 428 kb)
