# Supplementary material for: Coastal erosion—a “new” land-based source of labile mercury to the marine environment
Source: Environ Sci Pollut Res Int. 2018 Aug 10;25(28):28682–94. doi: 10.1007/s11356-018-2856-7 (PMC6153678; doi:10.1007/s11356-018-2856-7)
Supplement: Supplementary file 1 — (PDF 428 kb) [file 11356_2018_2856_MOESM1_ESM.pdf]

Supplementary material

The supplementary data to the article *Coastal erosion – a "new" land-based source of labile mercury to the marine environment* by U. Kwasigroch, M. Bełdowska, A. Jędruch and D. Saniewska (Institute of Oceanography, University of Gdańsk, Poland ✉ Corresponding author: urszula.kwasigroch@ug.edu.pl)

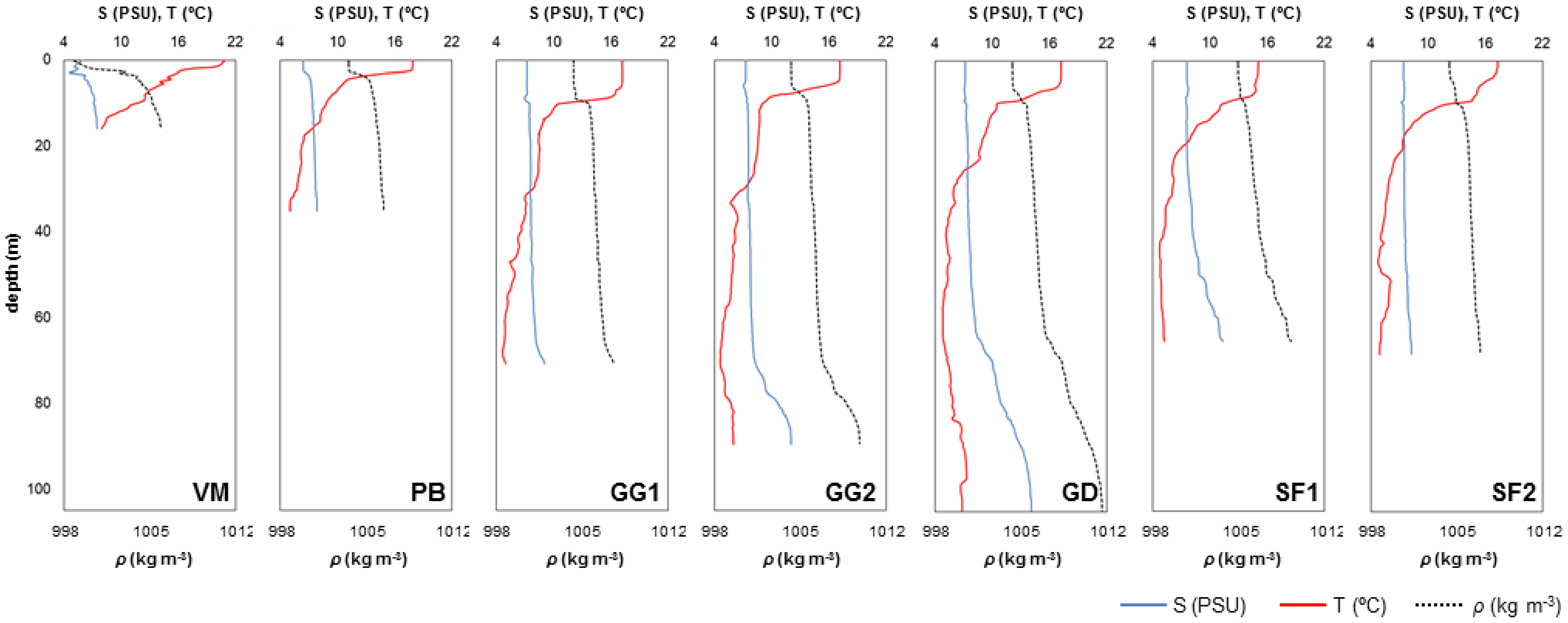

River outlets stations: ZR – Zagórska Struga River, RR – Reda River, GR – Gizdepka River, PR – Płutnica River
Offshore stations: VM – Vistula mouth, PB – Puck Bay, GG1, GG2 – central part of the Gulf of Gdańsk, GD – Gdańsk Deep, SF1, SF2 – Słupsk Furrow

Figure SI Depth profiles of basic sea water properties: temperature (T), salinity (S) and density (ρ) at offshore areas of the southern Baltic Sea

Table SI Median values and ranges of total mercury (Hg_{TOT}), labile (Hg_{ads1}, Hg_{abs}, Hg_{ads2}) and stable (HgS, Hg_{res}) mercury fractions in the sediments collected from the cliffs (horizontal and vertical cores) and in their area (beach, shallow coastal zone), as well as the median values of the basic sediment properties: content of organic matter (LOI), percentage of fine sediment fraction (FSF) and water content (W)

**station**	**layer (cm)**	**core orientation**	**Hg_{TOT} (ng g^{-1})**	**Hg_{ads1} (ng g^{-1})**	**Hg_{abs} (ng g^{-1})**	**Hg_{ads2} (ng g^{-1})**	**HgS (ng g^{-1})**	**Hg_{res} (ng g^{-1})**	**LOI (%)**	**FSF (%)**	**W (%)**
Orłowo cliff	0-20	horizontal	10.2 (9.6-12.8)	0.6 (0.3-0.9)	4.0 (3.5-5.4)	2.8 (2.1-3.3)	2.0 (1.7-2.8)	0.8 (0.7-0.9)	2.9 (2.8-3.1)	10.1 (9.8-10.3)	6.6 (6.2-7.1)
	0-20	vertical	29.6 (18.8-34.5)	1.9 (1.4-2.1)	22.6 (13.8-25.9)	0.5 (0.4-0.7)	3.8 (2.3-4.6)	0.8 (0.5-1.5)	8.4 (8.1-9.0)	24.9 (22.3-27.8)	8.5 (8.1-8.8)
	beach		0.9 (0.6-1.1)	0.1 (0.0-0.1)	0.4 (0.3-0.6)	0.1 (0.0-0.2)	0.1 (0.0-0.1)	0.2 (0.1-0.3)	0.5 (0.4-0.7)	0.0 (0.0-0.1)	0.3 (0.2-0.6)
	shallow coastal zone		1.5 (1.4-1.7)	0.3 (0.2-0.5)	0.7 (0.6-0.7)	0.1 (0.0-0.2)	0.3 (0.1-0.5)	0.1 (0.0-0.1)	0.8 (0.7-0.8)	0.2 (0.1-0.2)	18.1 (17.5-20.2)
Mechelinki cliff	0-20	horizontal	13.3 (11.8-13.9)	1.3 (1.0-1.4)	8.3 (7.2-9.4)	0.2 (0.1-0.2)	3.3 (3.1-3.4)	0.1 (0.0-0.1)	2.8 (2.7-3.0)	9.1 (8.5-10.2)	6.8 (6.0-7.5)
	20-40		8.9 (8.3-9.2)	1.0 (1.0-1.1)	5.5 (5.1-5.9)	0.3 (0.2-0.5)	2.0 (1.8-2.3)	0.2 (0.1-0.2)	1.9 (1.4-2.1)	7.2 (6.9-7.7)	6.4 (6.2-7.1)
	40-60		8.4 (8.1-8.5)	0.7 (0.6-0.9)	5.3 (5.1-5.4)	0.1 (0.0-0.1)	2.2 (2.0-2.4)	0.1 (0.1-0.3)	2.4 (2.3-2.6)	7.5 (6.8-7.6)	6.0 (5.8-6.1)
	0-20	vertical	25.4 (20.2-29.3)	1.8 (1.7-1.8)	14.0 (10.3-17.9)	1.8 (1.7-1.8)	7.7 (6.3-9.1)	0.1 (0.0-0.1)	6.4 (6.2-6.5)	20.8 (18.7-21.3)	9.2 (8.8-9.6)
	20-40		18.0 (13.1-32.0)	0.8 (0.6-1,4)	6.4 (4.1-13.1)	3.4 (2,6-5,5)	7.1 (5.5-14.6)	0.3 (0.2-0.5)	5.7 (5.5-5.9)	17.4 (16.1-22.9)	7.4 (7.1-7.6)
	40-60		19.5 (15.2-24.5)	1.0 (0.8-1.1)	7.0 (5.2-8.4)	4.1 (3.6-6.2)	6.9 (4.8-8.7)	0.5 (0.4-0.5)	5.1 (4.6-5.5)	14.2 (12.2-19.1)	6.3 (5.8-6.6)
	beach		1.3 (1.0-1.5)	0.2 (0.1-0.2)	0.6 (0.5-0.6)	0.1 (0.1-0.2)	0.2 (0.1-0.3)	0.2 (0.1-0.2)	0.4 (0.3-0.5)	0.1 (0.1-0.2)	0.5 (0.4-0.5)
	shallow coastal zone		2 (1.9-2.2)	0.4 (0.3-0.5)	0.8 (0.7-1.0)	0.2 (0.1-0.2)	0.5 (0.5-0.6)	0.0 (0.0-0.1)	1.3 (1.1-1.5)	0.9 (0.5-1.2)	16.8 (15.3-18.5)

Table SI Continued

station	layer (cm)	core orientation	Hg_{TOT} (ng g^{-1})	Hg_{ads1} (ng g^{-1})	Hg_{abs} (ng g^{-1})	Hg_{ads2} (ng g^{-1})	HgS (ng g^{-1})	Hg_{res} (ng g^{-1})	LOI (%)	FSF (%)	W (%)
Osłonino cliff	0-20	horizontal	10.5 (10.3-10.6)	1.4 (1.3-1.6)	3.6 (3.4-4.0)	1.6 (1.4-2.0)	3.5 (3.1-3.8)	0.4 (0.3-0.6)	2.2 (2.0-2.3)	11.1 (10.3-11.8)	7.8 (7.6-8.6)
	20-40		11.9 (10.6-12.3)	1.3 (1.2-1.6)	4.1 (3.8-4.3)	2.3 (2.0-2.7)	3.6 (3.1-3.8)	0.6 (0.4-0.7)	1.4 (1.2-1.5)	10.2 (9.4-10.5)	7.1 (6.6-7.5)
	40-60		8.3 (8.1-8.6)	0.6 (0.5-0.6)	2.5 (2.3-2.9)	1.5 (1.4-1.5)	3.3 (3.2-3.4)	0.5 (0.4-0.5)	1.3 (1.1-1.4)	9.1 (8.8-9.3)	5.1 (4.9-5.2)
	0-20	vertical	28.9 (26.0-32.1)	2.0 (1.7-2.2)	14.7 (12.6-16.9)	0.8 (0.7-0.9)	11.0 (9.8-14.2)	0.4 (0.3-0.5)	7.4 (6.2-8.1)	22.3 (19.9-24.2)	7.4 (7.1-7.9)
	20-40		17.1 (15.9-18.1)	0.8 (0.6-1.0)	7.3 (6.7-8.2)	1.1 (0.6-1.9)	7.4 (6.3-9.8)	0.5 (0.3-0.7)	3.8 (3.2-4.8)	19.5 (17.6-22.3)	5.3 (4.9-5.8)
	40-60		19.4 (14.4-24.0)	0.4 (0.3-0.5)	5.6 (3.8-8.9)	3.3 (2.1-4.6)	7.8 (6.1-10.6)	2.2 (1.8-2.4)	3.9 (3.6-4.1)	15.3 (14.2-16.4)	6.4 (6.0-6.8)
	beach		1.3 (1.0-1.5)	0.2 (0.1-0.3)	0.6 (0.5-0.7)	0.1 (0.1-0.2)	0.2 (0.1-0.2)	0.2 (0.1-0.2)	0.6 (0.4-0.7)	0.1 (0.0-0.1)	0.7 (0.6-0.8)
	shallow coastal zone		2.0 (1.7-2.1)	0.3 (0.2-0.4)	0.9 (0.8-1.2)	0.2 (0.1-0.2)	0.4 (0.3-0.5)	0.1 (0.0-0.1)	1.1 (1.0-1.1)	0.7 (0.6-0.8)	17.3 (15.9-18.5)
Puck cliff	0-20	horizontal	4.7 (3.7-5.6)	0.2 (0.1-0.3)	0.7 (0.6-0.9)	1.9 (1.5-2.2)	1.3 (1.0-1.6)	0.6 (0.5-0.8)	1.9 (1.5-2.2)	4.8 (4.5-5.1)	6.7 (6.5-7.0)
	20-40		5.1 (4.7-5.5)	0.2 (0.1-0.3)	0.7 (0.6-0.8)	2.4 (2.2-2.7)	1.1 (1.0-1.2)	0.7 (0.5-0.8)	1.3 (1.2-1.4)	4.1 (3.9-4.2)	6.4 (6.3-6.6)
	40-60		4.8 (4.4-5.4)	0.3 (0.2-0.5)	0.7 (0.5-0.9)	2.0 (1.9-2.2)	1.1 (1.0-1.2)	0.6 (0.4-0.7)	1.1 (1.0-1.3)	4.5 (4.4-4.7)	6.3 (6.1-6.6)
	0-20	vertical	21.6 (19.6-24.7)	1.3 (1.1-1.6)	11.9 (10.1-15.4)	0.3 (0.2-0.3)	7.9 (6.1-8.8)	0.2 (0.1-0.3)	5.3 (4.9-5.5)	19.4 (16.3-22.8)	13.3 (12.1-14.5)
	20-40		13.4 (8.7-19.4)	0.6 (0.3-0.7)	7.4 (4.8-12.9)	0.5 (0.3-0.6)	4.5 (2.9-5.2)	0.3 (0.2-0.3)	2.6 (1.8-3.2)	13.3 (9.2-16.1)	5.7 (4.2-6.8)
	40-60		7.9 (5.7-10.1)	0.3 (0.2-0.5)	3.8 (3.0-5.7)	0.5 (0.4-0.5)	2.9 (1.7-3.3)	0.4 (0.2-0.4)	1.8 (1.5-2.4)	10.1 (6.9-12.3)	5.1 (4.3-5.8)
	beach		0.9 (0.7-1.0)	0.1 (0.0-0.1)	0.4 (0.3-0.5)	0.2 (0.2-0.3)	0.1 (0.0-0.1)	0.1 (0.1-0.2)	0.4 (0.3-0.4)	0.0 (0.0-0.1)	0.4 (0.3-0.6)
	shallow coastal zone		1.6 (1.4-.1.8)	0.3 (0.3-0.4)	0.5 (0.4-0.6)	0.3 (0.2-0.3)	0.3 (0.2-0.4)	0.1 (0.1-0.2)	0.9 (0.7-1.0)	0.6 (0.4-0.7)	18.8 (16.7-19.8)

Table SII Median values and ranges of total mercury (Hg_{TOT}), labile (Hg_{ads1}, Hg_{abs}, Hg_{ads2}) and stable (HgS, Hg_{res}) mercury fractions in the surface sediments collected in river outlets and offshore areas of the southern Baltic Sea, as well as the median values of the basic sediment properties: content of organic matter (LOI), percentage of fine sediment fraction (FSF) and water content (W)

station	Hg_{TOT} (ng g^{-1})	Hg_{ads1} (ng g^{-1})	Hg_{abs} (ng g^{-1})	Hg_{ads2} (ng g^{-1})	HgS (ng g^{-1})	Hg_{res} (ng g^{-1})	LOI (%)	FSF (%)	W (%)
ZR	6.7 (4.5-8.1)	0.5 (0.2-0.7)	2.6 (1.8-3.6)	1.3 (1.1-1.5)	2.2 (1.2-2.5)	0.1 (0.0-0.1)	2.6 (2.1-3.4)	5.7 (4.5-6.2)	32.7 (28.5-35.7)
RR	7.8 (6.2-8.6)	0.5 (0.3-0.6)	2.7 (2.2-3.1)	1.8 (1.4-2.3)	2.8 (2.1-3.3)	0.1 (0.1-0.2)	4.2 (3.9-4.6)	4.6 (4.3-4.8)	39.8 (36.9-42.1)
GR	5.7 (5.3-6.2)	0.6 (0.4-0.8)	2.5 (2.3-2.7)	0.4 (0.3-0.6)	1.9 (1.7-2.2)	0.2 (0.1-0.2)	0.9 (0.8-1.1)	1.3 (1.1-1.5)	24.8 (22.9-26.8)
PR	3.6 (3.3-4.0)	0.3 (0.2-0.4)	1.6 (1.4-1.8)	0.6 (0.5-0.6)	1.1 (1.0-1.2)	0.1 (0.1-0.2)	0.7 (0.5-0.9)	0.9 (0.8-1.1)	22.5 (19.9-24.2)
VM	59.8 (57.5-61.4)	4.8 (4.7-5.0)	23.9 (21.3-25.2)	8.6 (8.3-8.9)	21.2 (20.6-22.3)	1.2 (1.0-1.3)	5.8 (5.7-6.0)	20.6 (19.9-21.0)	48.4 (47.4-49.8)
PB	153.8 (151.4-155.7)	5.3 (4.9-5.5)	88.0 (86.9-90.1)	24.9 (23.7-26.4)	33.6 (32.7-34.6)	2.1 (1.9-2.3)	8.6 (8.2-8.9)	38.0 (37.2-38.7)	69.6 (68.3-70.2)
GG1	130.5 (129.8-131.2)	3.3 (3.1-3.5)	99.8 (98.3-100.5)	18.5 (18.2-18.9)	8.4 (8.3-8.6)	0.4 (0.3-0.4)	14.3 (14.2-14.4)	35.2 (34.8-35.7)	66.1 (65.2-66.7)
GG2	116.9 (115.6-117.4)	3.6 (3.4-3.7)	76.8 (75.7-77.2)	12.8 (12.4-13.1)	23.0 (22.8-23.4)	1.1 (1.0-1.1)	12.4 (12.1-12.6)	34.1 (33.8-34.3)	62.6 (62.3-62.9)
GD	135.6 (134.8-136.3)	2.8 (2.7-2.9)	95.0 (94.6-95.7)	20.7 (20.3-20.9)	15.3 (15.1-15.6)	1.7 (1.6-1.8)	12.3 (12.1-12.5)	15.9 (15.8-16.1)	71.1 (70.5-71.6)
SF1	9.7 (9.4-9.9)	0.8 (0.7-0.9)	4.6 (4.4-4.8)	1.0 (0.9-1.1)	3.1 (2.9-3.2)	0.2 (0.1-0.2)	1.2 (1.1-1.3)	5.3 (5.1-5.4)	27.3 (27.0-27.6)
SF2	7.1 (7.0-7.2)	0.8 (0.7-0.9)	4.2 (4.1-4.3)	0.4 (0.4-0.5)	1.6 (1.4-1.7)	0.1 (0.1-0.2)	1.7 (1.6-1.7)	1.9 (1.8-2.0)	26.8 (26.1-27.2)

River outlets stations: ZR – Zagórska Struga River, RR – Reda River, GR – Gizdepka River, PR – Płutnica River
Offshore stations: VM – Vistula mouth, PB – Puck Bay, GG1, GG2 – central part of the Gulf of Gdańsk, GD – Gdańsk Deep, SF1, SF2 – Słupsk Furrow

Table SIII Relationships between total mercury concentration (Hg_{TOT}), content of organic matter (LOI) and fine fraction (FSF) in sediments collected from the cliffs and at offshore areas of the southern Baltic Sea (Spearman's rank correlation coefficients)

sediment type			Hg_{TOT} (ng g^{-1})	FSF(%)	LOI (%)
cliff	vertical cores	**Hg_{TOT} (ng g^{-1})**		0.88	0.79
		**FSF (%)**	0.88		0.85
		**LOI (%)**	0.79	0.85	
	horizontal cores	**Hg_{TOT} (ng g^{-1})**		0.70	0.60
		**FSF (%)**	0.70		0.51
		**LOI (%)**	0.60	0.51	
marine	surface sediment	**Hg_{TOT} (ng g^{-1})**		0.71	0.61
		**FSF (%)**	0.71		0.60
		**LOI (%)**	0.61	0.60	

Red coefficients are statistically significant ($p < 0.05$)
